# Comparison of neuromuscular blockade recovery co-administered with neostigmine and different doses of calcium gluconate: a randomized control trial

**DOI:** 10.1186/s12871-021-01316-7

**Published:** 2021-03-29

**Authors:** So Ron Choi, Jeong Ho Kim, Kyung Hyun Lee, Sang Yoong Park

**Affiliations:** Department of Anesthesiology and Pain Medicine, Dong-A University Hospital, 26, Daesingongwon-ro, Seo-gu, Busan, 49201 Republic of Korea

**Keywords:** Acetylcholinesterase inhibitor, Calcium gluconate, Neostigmine, Neuromuscular blockade recovery, Postoperative residual curarization

## Abstract

**Background:**

Calcium increases the probability of transmitter release at the neuromuscular junction. It is not known whether there is a dose-dependent relationship between the dosage of calcium gluconate and the probability of transmitter release for non-depolarizing neuromuscular blockade (NMB) recovery by acetylcholinesterase inhibitors (AchEIs). This study compared the neuromuscular recovery time and the incidence of postoperative residual curarization (PORC) according to the dosage of calcium gluconate co-administered with neostigmine in three patient groups.

**Methods:**

Patients were randomly allocated to a control group, a 5 mg/kg calcium gluconate group (calcium 5 group), or a 10 mg/kg calcium gluconate group (calcium 10 group). In patients with a TOF ratio (TOFr) between 0.2–0.7, 0.04 mg/kg of neostigmine was administered and both 0.2 mg of glycopyrrolate and 0.4 mg of atropine per 1 mg of neostigmine were administered. And additional 5 or 10 mg/kg of calcium gluconate were administrated to the calcium 5 and 10 groups. The primary endpoint was neuromuscular recovery time (the time between reversal and TOFr≥0.9). The secondary endpoints were the incidence of PORC at 5, 10, and 20 min after reversal administration and the train-of-four ratio (TOFr) at each time point.

**Results:**

The neuromuscular recovery time was 5.3 min in the control group, 3.9 min in the calcium 5 group, and 4.1 min in the calcium 10 group, respectively (*P* = 0.004). The incidence of PORC at 5 min after neostigmine administration was 12 in the control group, 4 in the calcium 5 group, and 4 in the calcium 10 group, respectively, with statistical significance (*P* = 0.014).

**Conclusions:**

The co-administration of calcium gluconate with neostigmine safely promoted early NMB recovery, and the neuromuscular recovery time of the calcium 10 group tended to be more evenly distributed than that of the calcium 5 group.

**Trial registration:**

https://cris.nih.go.kr/cris/index.jsp(KCT0004182). Date of registration: August 122,019.

## Background

It has been well-reported that the release of acetylcholine (Ach) at the neuromuscular junction is calcium-dependent [1]. Calcium increases the probability of transmitter release at the neuromuscular junction. A brief calcium administration restrictively and immediately before the depolarizing phase accelerates transmitter release [2]. However, the overall details of the role of ionized calcium for neuromuscular blockade (NMB) recovery by acetylcholinesterase inhibitors (AchEI) have not yet been clarified [3].

AchEIs such as neostigmine are widely used clinically for non-depolarizing NMB recovery. Neostigmine inhibits acetylcholinesterase and increases the Ach concentration at the neuromuscular junction to achieve neuromuscular function recovery. However, in addition to the pharmacodynamics between non-depolarizing neuromuscular blocking agents (NMBAs) and AchEIs, factors such as electrolyte abnormalities, anticholinesterase administration timing, body temperature, drug dosage, additional medications, and liver or kidney disease also affect neuromuscular recovery [4]. Therefore, it is essential for anesthesiologists to take these various factors into consideration when recovering NMB.

To determine whether ionized calcium at different doses may have a quantitative effect, this study compared the neuromuscular recovery time and the incidence of postoperative residual curarization (PORC) according to the dosage of calcium gluconate co-administered with neostigmine in three patient groups: a 5 mg/kg calcium gluconate group (calcium 5 group), a 10 mg/kg calcium gluconate group (calcium 10 group), and a control group.

## Methods

### Study design and patient allocation-

This single-center, randomized, controlled, double-blind, parallel-group trial was approved by the Institutional Review Board of Dong-A University Hospital (IRB No. DAUHIRB -19-127, 3 July 2019). This trial was registered with Korean Clinical Trials at cris.nih.go.kr (KCT0004182; August 12, 2019).

Patients who met the following inclusion criteria were selected between August 2019 and June 2020: American Society of Anesthesiologists physical status classes I-III, 20–70 years of age, body mass index (BMI) of 18.5–25, body weight of 50–75 kg, and scheduled elective lower abdomen laparoscopic surgery with an expected duration of at least 120 min under general anesthesia. Among these patients, those who had normal magnesium and calcium concentrations during the preoperative blood test and just before emergence from anesthesia were targeted for the study. Written informed consent was obtained prior to study participation. The exclusion criteria included the following: a history of neuromuscular disease or hepatic and renal disease, pregnancy, use of medications known to influence the potency or duration of NMBAs, breastfeeding, a history of malignant hyperthermia and hyper−/hypocalcemia, allergy to medications used in this study, or having contraindications to atropine or neostigmine. If two or more types of cooperative surgery were performed, or if the patient was admitted to the intensive care unit (ICU) after surgery, then those with levels beyond the normo-calcemic range during the blood test immediately before emergence from anesthesia or during conversion from laparoscopic to open surgery were eligible for dropout during the study.

Patients were randomly allocated to the control, calcium 5, or calcium 10 group in a double-blinded manner. The randomization table was archived by an unrelated investigator to ensure allocation concealment. Medications were prepared by an anesthesia nurse who was not part of the study. The total amount of all study drugs was adjusted to 15 mL by adding normal saline, and the study operators were blinded to the study medications.

Patients were monitored using non-invasive and invasive arterial blood pressure devices and a pulse oximeter. They underwent electrocardiography (EKG) and bispectral index (BIS) analysis. Anesthesia was induced with a target-controlled infusion device (Orchestra Infusion Workstation; Fresenius Vial, Brezins, France), by infusion of propofol and remifentanil (remifentanil at an effect-site concentration of 4.0 ng/mL, followed by propofol at an effect-site concentration of 4.0 μg/mL). Each drug was titrated to maintain the mean arterial pressure within ±20% of baseline and the BIS at 30–60.

After loss of consciousness and before administration of rocuronium, quantitative continuous neuromuscular monitoring using acceleromyography (TOFscan®, IdMed; Marseille, France) was performed to assess the response of the adductor pollicis to ulnar nerve stimulation [5]. The acceleration transducer was placed at the volar side of the distal thumb. The forearm and fingers were immobilized and disinfected, and the stimulation electrodes were placed on the clean skin over the ulnar nerve close to the wrist [6]. Acceleromyography was calibrated via the automated calibration mode, with at least 2 min to stabilize the TOF stimulation prior to NMBA administration. After the calibration had stabilized, patients were administered 0.8 mg/kg of rocuronium. The TOF response was monitored at a frequency of 2 Hz of 200 ms, for a duration of 1.5 s, every 15 s [7]. The current intensity was 50 mA, and the average of the two TOF measurements at 15 s intervals was recorded. If the measurements differed by > 10%, additional TOF measurements were performed, and the closest two values were averaged [8]. When the TOF count (TOFc) reached 0, endotracheal intubation was performed. Mechanical ventilation (tidal volume 5–7 mL/kg) was administered to retain an end-tidal CO_2_ of 35–40 mmHg. An additional 0.02 mg/kg of vecuronium was administered when the TOFc was at least 2. NMBAs were not administered during the last 30 min of the surgery. The patients were warmed to maintain a core body temperature of > 35.0 °C and < 37.0 °C using an air-circulating heating blanket.

By the end of the operation, a spontaneous NMB recovery was achieved when the TOFc returned to 4. In patients with a TOF ratio (TOFr) between 0.2–0.7, 0.04 mg/kg of neostigmine was administered and both 0.2 mg of glycopyrrolate and 0.4 mg of atropine per 1 mg of neostigmine were administered. And additional 5 or 10 mg/kg of calcium gluconate were administrated to the calcium 5 and 10 groups [6]. The patients were extubated and transported to the post-anesthesia care unit (PACU) after adequate recovery, defined as an achievement of TOFr ≥0.9 [4]. When patients responded to instructions, cooperated, and complained of pain, they were administered 1 μg/kg of fentanyl for postoperative analgesia.

It was necessary to study whether there was a significant difference in hemodynamic change between groups according to the dose of administrated calcium gluconate. To analyze hemodynamic changes, the blood pressure (BP) and heart rate (HR) were analyzed immediately before, immediately after, and 10 min after administration of the reversal agent. Changes in serum calcium concentration before and after calcium gluconate administration were monitored through blood tests just before and 20 and 40 min after administration of the reversal agent. Moreover, close EKG monitoring was conducted to ensure safety. Pulmonary spirometry was performed before the operation and 20 min after reversal. A pulmonary function test consisted of forced expiratory volume in 1 s (FEV1) and forced expiratory volume in 1 s/forced vital capacity (FEV1/FVC). Respiratory function measurement was conducted using a spirometer (Micro I handheld spirometer; CareFusion, Yorba Linda, CA) at the PACU. All patients were positioned in a chair with the knee flexed (20–30°) and the upper body raised (30°). As patient cooperation is essential in obtaining valid spirometric measurements, the patients were trained so as to maximize their respiratory effort. A total of three attempts were made, and the average of the three values was used.

### Outcomes

The primary endpoint was neuromuscular recovery time (the time between reversal and TOFr≥0.9). The secondary endpoints were the incidence of PORC at 5, 10 and 20 min after reverse administration and the TOFr at each time point. PORC was defined as having a TOFr < 0.9 [6]. The secondary outcome also included pulmonary function tests and blood tests. A PACU nurse who did not participate in the study checked for symptoms such as pharyngeal dysfunction, swallowing difficulty, and airway obstruction requiring jaw thrust, indicating PORC [6, 9].

### Statistical analysis

The results were obtained through per-protocol analysis. The sample size was determined based on neuromuscular recovery time. Using neostigmine, the usual neuromuscular recovery time was 500 ± 200 s [10]. Considering that the neuromuscular recovery time decreased by about 250 s when 10 mg/kg calcium gluconate was co-administered with neostigmine, a group size of 23 was required to discriminate statistically significant differences with a type I error of 0.05 and a power of 0.8. Assuming a dropout rate of 20%, the number of patients required per group was 28.

Data are expressed as medians with interquartile ranges (IQRs) or numbers with proportions (%), as appropriate. The normality of continuous data distribution was evaluated through the Shapiro-Wilk test. Continuous variables were analyzed by analysis of variance or the Kruskal-Wallis test, as appropriate. Categorical variables were analyzed using Fisher’s exact test. Box plots were used for comparison between groups. Bonferroni’s correction was performed for all multiple comparisons, and *p*-values < 0.017 were considered statistically significant. Statistical analysis was performed using SPSS 26 (IBM, Armonk, NY).

## Results

### General characteristics of the enrolled subjects

**Fig. 1:**
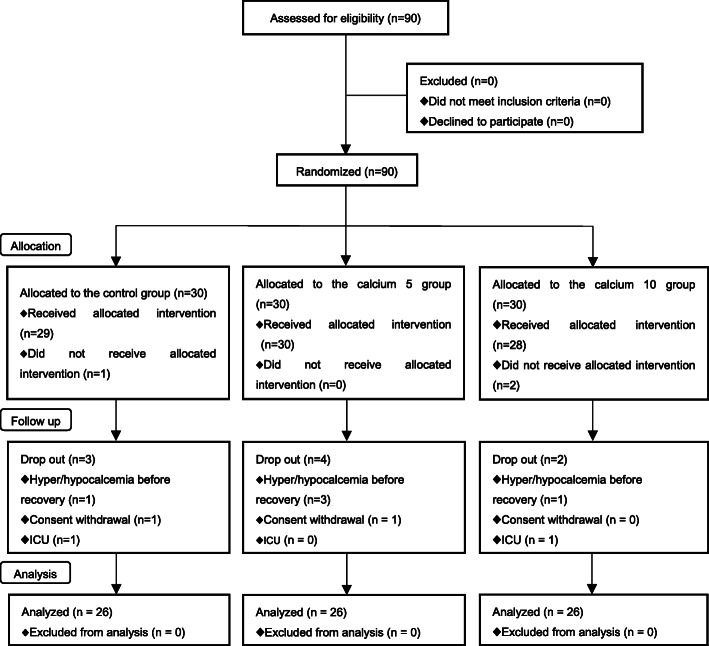
CONSORT diagram. Flowchart describing patient allocation and analysis

A total of 90 patients were recruited for this trial. They were divided into 3 groups: the control group (*n* = 30), calcium 5 group (*n* = 30), and calcium 10 group (*n* = 30). Due to various reasons for dropout, 4 from each group (about 13%) were eliminated. Twelve patients were excluded from analysis after randomization because of hyper−/hypocalcemia prior to emergence from anesthesia, consent withdrawal, or ICU admission after surgery (Fig. 1). Finally, 78 patients were included in the analysis. Differences in patient characteristics, preoperative serum calcium and magnesium concentrations, and intraoperative data were not significant among the three groups (Table 1).

**Table 1:** Baseline patient characteristics

Parameters	Control group	Calcium 5 group	Calcium 10 group	P
n	26	26	26	
Age (years)	55 (48 to 59)	59 (53 to 62)	57 (54 to 60)	0.790
Male	20	14	21	0.071
Height (cm)	166.7 (159.6 to 173.7)	162.8 (157.5 to 169.6)	168.0 (158.5 to 171.6)	0.201
Weight (kg)	65.6 (63.2 to 67.9)	64.2 (52.8 to 71.5)	63.4 (55.0 to 69.5)	0.431
BMI (kg/m^2^)	24.1 (22.6 to 24.9)	23.6 (21.4 to 25.4)	23.3 (21.1 to 24.1)	0.196
Preoperative serum calcium concentration (mg/dL)	4.4 (4.3 to 4.7)	4.5 (4.3 to 4.5)	4.5 (4.3 to 4.6)	0.964
Preoperative serum magnesium concentration (mg/dL)	2.2 (2.1 to 2.2)	2.1 (1.9 to 2.2)	2.1 (2.0 to 2.1)	0.052
Temperature at neostigmine administration (°C)	35.9 (35.6 to 36.3)	36.0 (35.5 to 36.2)	36.0 (35.8 to 36.3)	0.511
Total dose of rocuronium (mg)	50 (50 to 60)	50 (50 to 60)	50 (50 to 60)	0.810
Total dose of vecuronium (mg)	6 (4 to 6)	7 (6 to 8)	7 (5 to 9)	0.120
Duration of surgery (min)	135 (120 to 155)	138 (130 to 160)	140 (125 to 175)	0.909
Duration of anesthesia (min)	195 (160 to 215)	190 (180 to 215)	185 (175 to 240)	0.776

Data are presented as median (Q1 to Q3) or number of patients. *BMI* Body mass index, *FEV1* Forced expiratory volume in 1 s, *FVC* Forced vital capacity, calcium 5 group, 5 mg/kg calcium gluconate group; calcium 10 group, 10 mg/kg calcium gluconate. *P* < 0.017 as statistical significance

### Comparison of neuromuscular recovery time

The TOFr at reversal agent administration was [median (Q1-Q3)] 0.50 (0.35–0.61) in the control group, 0.51 (0.44–0.60) in the calcium 5 group, and 0.47 (0.43–0.62) in the calcium 10 group, respectively (*P* = 0.855). The neuromuscular recovery time (minute), primary end point, was 5.3 (3.9–6.9) in the control group, 3.9 (2.9–4.1) in the calcium 5 group, and 4.1 (2.1–4.9) in the calcium 10 group, respectively (*P* = 0.004).

**Fig. 2:**
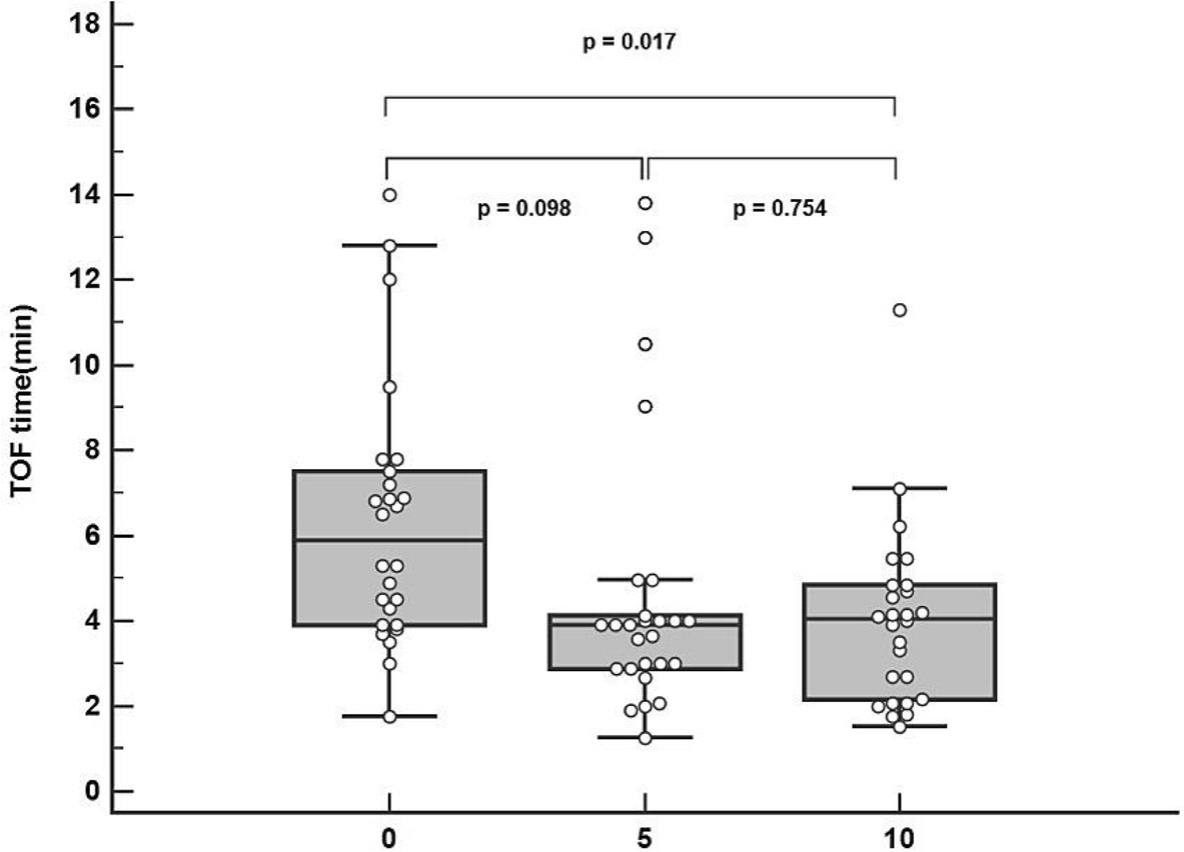
Boxplot showing the neuromuscular blockade recovery time of the three groups. Each dot represents the measurement for each patient. X-axis: 0, control group; 5, calcium 5 group; 10, calcium 10 group. Y-axis: neuromuscular recovery time (min) to reach a TOFr of 0.9. *P*-values were obtained by comparing each of the two groups. The standard deviation was the smallest in the calcium 10 group and outliers were the most prevalent in the calcium 5 group. *P* < 0.017 as statistical significance. TOF, train-of-four; TOFr, train-of-four ratio

Figure 2 is a box plot showing the results of neuromuscular recovery time in the three groups, and the *p* values analyzed by each of the two groups were added. The neuromuscular recovery time was shorter in the calcium 10 group than in the control group with no statistical significance (*P* = 0.017). There was not a statistical difference between the control and calcium 5 groups (*P* = 0.098) or between the calcium 5 and calcium 10 groups (*P* = 0.754).

### Comparison of incidence of PORC and TOFr at each timepoint

Table 2 shows the incidence of PORC and TOFr after neostigmine administration at each time point. The incidence of PORC at 5 min after neostigmine administration was 12 in the control group, 4 in the calcium 5 group, and 4 in the calcium 10 group, respectively, with statistical significance (*P* = 0.014). There were no significant differences among the three groups in terms of PORC incidence at 10 and 20 min after neostigmine administration. There were also no significant differences in TOFr at 5, 10, and 20 min after neostigmine administration among the three groups. All patients were extubated after surgery. There was no significant difference in the time required for extubation and end of anesthesia in the three groups. None of the patients showed clinical signs of PORC upon leaving the PACU.

**Table 2:** Neuromuscular blockade recovery after neostigmine administration

Group	Control group	Calcium 5 group	Calcium 10 group	P
n	26	26	26	
Incidence of PORC after neostigmine administration				
5 min	12	4	4	0.014
10 min	4	4	1	0.374
20 min	0	0	0	
TOFr after neostigmine administration				
5 min	0.87 (0.82 to 0.93)	0.92 (0.90 to 0.93)	0.93 (0.90 to 0.96)	0.098
10 min	0.93 (0.93 to 0.97)	0.94 (0.92 to 0.96)	0.96 (0.92 to 0.99)	0.421
20 min	1.00 (0.99 to 1.00)	1.00 (0.98 to 1.00)	1.00 (0.99 to 1.00)	0.477
Time from neostigmine administration to				
Extubation (min)	8.6 (6.0 to 10.1)	7.9 (6.6 to 8.1)	8.2 (7.0 to 8.6)	0.885
End of anesthesia (min)	9.6 (8.0 to 11.5)	8.8 (7.4 to 12.0)	9.0 (8.7 to 9.5)	0.483

Data are presented as median (Q1 to Q3) or number of patients. *PORC* Postoperative residual curarization, *TOFr* Train-of-four ratio; calcium 5 group, 5 mg/kg calcium gluconate group; calcium 10 group, 10 mg/kg calcium gluconate group. *P* < 0.017 as statistical significance

### Comparison of hemodynamic changes at each timepoint

**Fig. 3:**
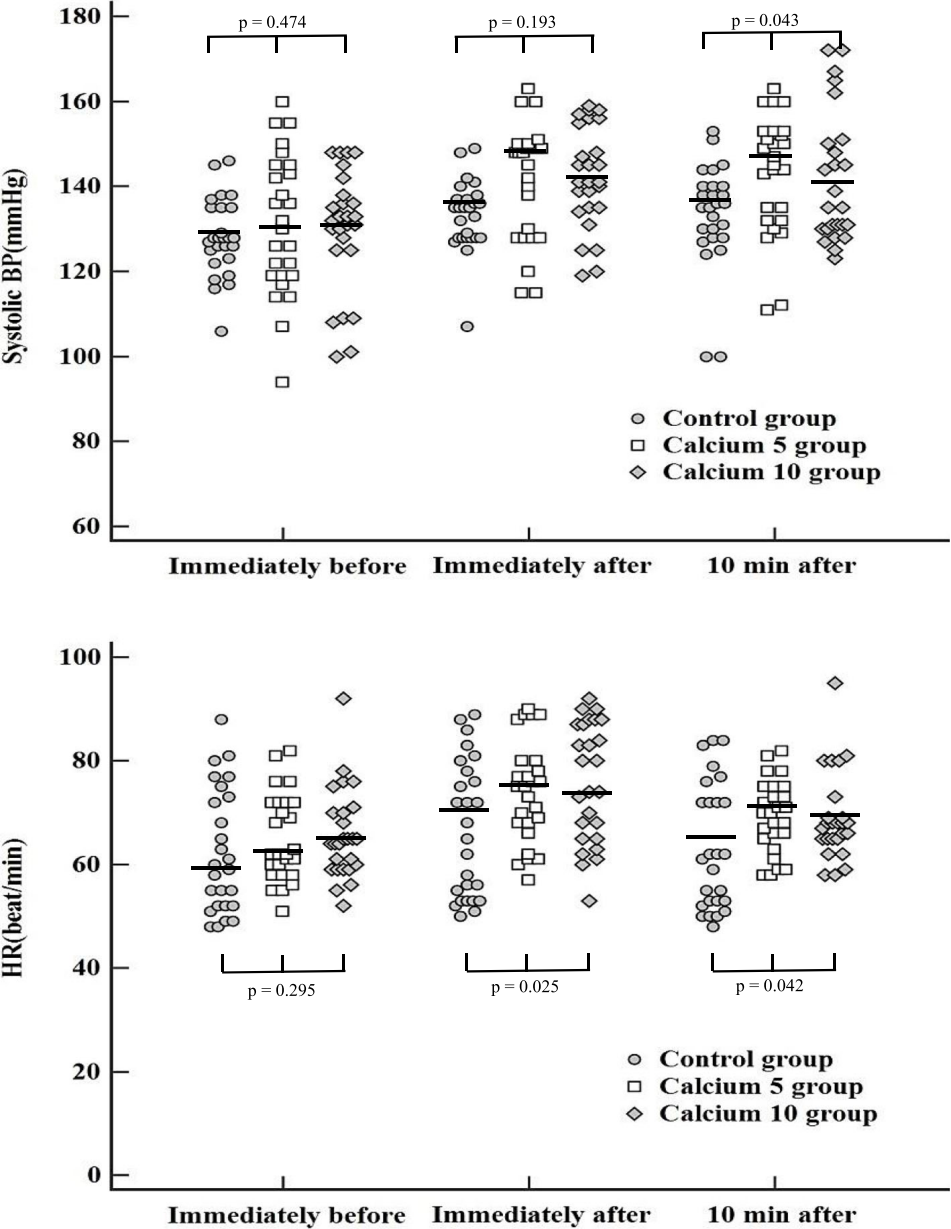
Hemodymic changes at each timepoint. Each dot represents the measurement for each time point. X-axis: immediately before, immediately after and 10 min after neostigmine administration. Y-axis: systolic Blood Pressure (mmHg), Heart Rate (beat/min) of all patients at each time point. P-values were obtained by comparing each of the three groups. The thick straight line is the median value of each group. BP, blood pressure; HR, heart rate; TOF, train-of-four; TOFr, train-of-four ratio

Systolic BP and HR were analyzed immediately before, immediately after, and 10 min after reversal agent administration. Figure 3 shows the hemodynamic values measured at each timepoint. In all groups, a transient hyperdynamic state was induced immediately after drug administration, but they were within a stable range of ±20% compared to the values measured immediately before as a baseline. After 10 min, the hemodynamic indicators of all groups were also within the stable range. There were no significant differences between groups at any timepoint.

### Perioperative serum ionized calcium concentrations and pulmonary function test

In this study, serum ionized calcium concentrations were obtained 20 and 40 min after neostigmine administration (Table 3). None of the patients developed hypercalcemia, and all data were within the range of normo-calcemia. No specific findings were observed on EKG monitoring.

**Table 3:** Perioperative calcium concentration (mg/dL)

Groups	Control group	Calcium 5 group	Calcium 10 group	P
n	26	26	26	
Calcium concentration at neostigmine administration (mg/dL)				
Just before	4.3 (4.1 to 4.6)	4.3 (4.2 to 4.6)	4.4 (4.2 to 4.6)	0.828
After 20 min	4.3 (4.1 to 4.5)	4.3 (4.3 to 4.4)	4.5 (4.2 to 4.7)	0.132
After 40 min	4.1 (3.8 to 4.3)	4.1 (3.9 to 4.3)	4.1 (3.9 to 4.4)	0.603

Data are presented as median (Q1 to Q3). Data on calcium concentration were obtained before and 20 and 40 min after neostigmine administration. Calcium 5 group, 5 mg/kg calcium gluconate group; calcium 10 group, 10 mg/kg calcium gluconate group. *P* < 0.017 as statistical significance

None of the groups demonstrated significant differences in FEV1 and FEV1/FVC before surgery and 20 min after administration of reversal agent. Changes in FEV1 (%) before surgery and 20 min after reversal were − 9.2 (− 13.4 to 3.0) in the control group, − 11.7 (− 33.1 to − 3.6) in the calcium 5 group, and − 4.2 (− 18.2 to 7.0) in the calcium 10 group, with no significance (*P* = 0.146). Changes in FEV1/FVC (%) before surgery and 20 min after reversal were − 0.8 (− 4.6 to 2.5) in the control group, − 1.0 (− 3.6 to 2.1) in the calcium 5 group, and 0.8 (− 3.5 to 2.9) in the calcium 10 group, with no significance (*P* = 0.699).

## Discussion

This study was designed to examine the effectiveness of calcium administration, reversing NMB with neostigmine. If the complete recovery of neuromuscular function is not achieved during emergence from anesthesia, PORC may occur, leading to dangerous clinical outcomes such as hypoxemic event, airway obstructions, reduced airway volumes, and postoperative pulmonary complications [8]. It is significant to conduct neuromuscular monitoring to reduce such risk. Ju et al. [11] demonstrated that when NMB was reversed with neostigmine co-administered with 5 mg/kg of calcium chloride, the neuromuscular recovery time was 25% shorter than that of the control group without calcium chloride. Through this, it was found that ionized calcium was one of the factors influencing neuromuscular recovery. In this study, the neuromuscular recovery time was 22.6% shorter in the calcium 10 group compared to the control group with borderline significance (*P* = 0.017). The neuromuscular recovery time of the calcium 10 group tended to be more evenly distributed than that of the calcium 5 group, with a lower standard deviation. The incidence of PORC at the early neuromuscular recovery period was lowest in the calcium 10 group. So our principal finding was that administration of 10 mg/kg of calcium gluconate with neostigmine would safely promote early NMB recovery and expected to be more effective in obtaining the desired result of enhancing early neuromuscular recovery, compared with both control group and calcium 5 group.

AchEIs and sugammadex reverse neuromuscular blocks by completely different mechanisms. AchEIs such as neostigmine act through an indirect competitive antagonistic mechanism with nondepolarizing NMBAs. Sugammadex is a synthetically modified g-cyclodextrin with a hydrophobic core and a hydrophilic exterior, designed to encapsulate steroidal NMBAs. Sugammadex more rapidly reverses the NMB than AchEI and less gastrointestinal side effect [12]. Anesthesiologists in countries where sugammadex is available may less concerned about the disadvantages of AchEIs such as recurarization or bradycardia, postoperative nausea and vomiting secondary to stimulation of muscarinic cholinergic receptors. Although there are many advantages of sugammadex, usage of AchEIs cannot disappear, and the study of how to take AchEIs more useful and what factors interact with AchEIs seems to remain indispensable.

Sometimes there are situations in which AchEIs should be administered rather than sugammadex. First, AchEIs are never completely replaceable for a variety of reasons, including the limited usage of sugammadex, in patients with impaired renal function. The current safety of sugammadex is not yet established in patients with severe renal function impairment [13]. Second, sugammadex binds only to steroidal NMBAs, and AchEIs reverse NMB induced by both steroidal and benzylisoquinolinium NMBAs. Therefore AchEIs are the only effective agent against benzylisoquinolinium NMBAs [14]. In situations such as re-surgery or re-intubation immediately after sugammadex reversal, the use of benzylisoquinolinium NMBAs is recommended to obtain new NMB in most countries [15]. Third, the cost of sugammadex should also be considered. In our country, sugammadex is 105 times more expensive than neostigmine (105 $ vs 1 $). Therefore, anesthesiologists should take into account the economic circumstances of the patient of their health. If there was a reappearance of a TOFc of 4, the sugammadex 1 vial confers a little economic advantage for AchEIs because it can also achieve NMB recovery within 5 min when calcium is coadministrated [10]. Finally, neuromuscular recovery should be performed with AchEIs in patients with a history of sugammadex anaphylaxis. The incidence of sugammadex anaphylaxis is about 0.03–0.098%, where some cases did not show an anaphylatic reaction to neostigmine [16, 17]. Patients with a history of rocuronium anaphylaxis were likely to cross-positive for succinylcholine (44%) and vecuronium (40%). Cisatracurium had the lowest chance of cross-reactivity of 5% [18]. While sugammadex has many advantages over the classic reversal agents, AchEIs, the disadvantages of neostigmine are acceptable compared to the lethal risk of anaphylaxis caused by sugammadex.

The total serum calcium level is maintained between 8.5–10.5 mg/dL by homeostatic equilibrium. Symptoms of hypercalcemia appear when the serum calcium concentration is about 15 mg/dL. With the administration of 10 mg/kg of calcium gluconate, the serum calcium concentration increases by 1.4 mg/dL [19]; thus, 10 mg/kg of calcium gluconate can be safely used in normo-calcemic patients. In this study, 5 and 10 mg/kg of calcium gluconate were added and a slight increase in ionized calcium concentration was induced, but there was no occurrence of significant hypercalcemia in any groups.

There is a positive correlation between total calcium and ionized calcium [20]. It is the ionized form of calcium that affects the probability of transmitter release at the neuromuscular junction. The rapid rise of ionized calcium instantly antagonizes the sensitivity, that is neuromuscular blocking effects of NMBAs, which are potentiated when it rapidly decreases [21]. However, this interactions by ionized calcium were statistically significant but minor clinical significance, which might be related to our principal findings.

When calcium gluconate or usual reversal agents were administrated, hemodynamic changes could often be induced. It was important to closely observe the hemodynamic changes in all groups because they may be influenced by one or both factors. Immediately after reversal agent administration, all three groups transiently became hyperdynamic within a safe range of 20%, and 10 min after that, there were no significant differences between the three groups. A transient hyperdynamic state in early neuromuscular recovery might be caused by the addition of atropine and glycopyrrolate [22]. Shapira et al. [23] demonstrated that when calcium chloride was administered, early hemodynamic changes were observed within 20 s, and the cardiac index returned to baseline after about 1 min. This might be related to the transient hyperdynamic state only in the early period of NMB reversal and the lack of significant differences at the time of late neuromuscular recovery. It might have induced a synergistic effect with the AchEIs and ionized calcium by the rapid exchange of ionized calcium in contractile cell membranes of the heart [24].

Some studies investigated the overall action of ionized calcium on synapses, demonstrating that ionized calcium facilitates transmitter release or contributes in part to synaptic delay [2]. When calcium channel blockers are administered, spontaneous presynaptic Ach release is diminished, and vecuronium binding at the postsynaptic receptor is delayed due to the reduced release of presynaptic Ach [25]. Conversely, in patients with hyperparathyroidism and severe hypercalcemia, there was an antagonistic effect on vecuronium where the onset was slower and the duration was shorter than in patients with normo-calcemia [26]. Taking these points into consideration, calcium seems to have an effect against NMB. Thus calcium administration would help facilitate recovery from NMB by non-depolarizing NMBAs. The method of adding calcium to the classic reversal agent is not yet recommended over the past decades, but it has been found that in the shallow NMB state, adding calcium can accelerate recovery.

This study has some limitations. First, patients with liver and kidney diseases, which comprise the majority of cases requiring AchEIs, were excluded from this study. Hence, further research is required to investigate the safety and efficacy of neuromuscular recovery using calcium in patients with these conditions. Second, this study did not demonstrate the pharmacodynamics of ionized calcium combined with AchEIs, which might be related to these findings. Further study is warranted to elucidate the pharmacodynamics or mechanism of action of combined administration of calcium gluconate and AchEIs at the neuromuscular junction. Third, the results were only applicable for shallow NMB. If the deep NMB is present, the full recovery time using neostigmine is above 1 h [14]. Therefore, further study is needed on the effect of ionized calcium administered with neestigmine in deep NMB. Fourth, what results might be in the case of different doses of neostigmine needs to be studied.

## Conclusions

The co-administration of calcium gluconate with neostigmine safely promoted early NMB recovery, and the neuromuscular recovery time of the calcium 10 group tended to be more evenly distributed than that of the calcium 5 group. Therefore it is expected to be more effective in obtaining the desired result of enhancing early neuromuscular recovery than control group and calcium 5 group.

## Data Availability

Due to personal information issues, it cannot be provided collectively. It is being stored in the hospital data server. We will provide it separately if there is a later request. The raw data of the current study are available from the corresponding author on request.

## Abbreviations

calcium 5 group: 5 mg/kg calcium gluconate group
calcium 10 group: 10 mg/kg calcium gluconate group
Ach: Acetylcholine
AchEI: Acetylcholinesterase inhibitor
BP: Blood pressure
CCBs: Calcium channel blockers
FEV1: Forced expiratory volume in 1 s
FVC: Forced vital capacity
HR: Heart rate
NMB: Neuromuscular blockade
NMBAs: Non-depolarizing neuromuscular blocking agents
PACU: Post-anesthesia care unit
PORC: Postoperative residual curarization
TOFc: TOF count
TOF: Train-of-four
TOFr: Train-of-four ratio
